# Care of Bullet-related Injuries: A Cross-sectional Study of Instructions and Prescriptions Provided on Discharge from the Emergency Department

**DOI:** 10.5811/westjem.2022.11.57574

**Published:** 2023-02-27

**Authors:** Jane M. Hayes, L.J. Punch, Kristen L. Mueller

**Affiliations:** * Washington University School of Medicine, St. Louis, Missouri; † The T, St. Louis, Missouri; ‡ Washington University School of Medicine, Department of Emergency Medicine, St. Louis, Missouri

## Abstract

**Introduction:**

There are more than 80,000 emergency department (ED) visits for non-fatal bullet-related injuries (BRI) per year in the United States. Approximately half of these patients are discharged home from the ED. Our objective in this study was to characterize the discharge instructions, prescriptions, and follow-up plans provided to patients discharged from the ED after BRI.

**Methods:**

This was a single-center, cross-sectional study of the first 100 consecutive patients who presented to an urban, academic, Level I trauma center ED with an acute BRI beginning on January 1, 2020. We queried the electronic health record for patient demographics, insurance status, cause of injury, hospital arrival and discharge timestamps, discharge prescriptions, and documented instructions regarding wound care, pain management, and follow-up plans. We analyzed data using descriptive statistics and chi-square tests.

**Results:**

During the study period, 100 patients presented to the ED with an acute firearm injury. Patients were predominantly young (median age 29, interquartile range 23–38 years), male (86%), Black (85%), non-Hispanic (98%), and uninsured (70%). We found that 12% of patients did not receive any type of written wound care instruction, while 37% received discharge paperwork that included instructions to take both an NSAID and acetaminophen. Fifty-one percent of patients received an opioid prescription, with a range from 3–42 tablets (median 10 tablets). The proportion of patients receiving an opioid prescription was significantly higher among White patients (77%) than among Black patients (47%).

**Conclusion:**

There is variability in prescriptions and instructions provided to survivors of bullet injuries upon ED discharge at our institution. Our data indicates that standardized discharge protocols could improve quality of care and equity in the treatment of patients who have survived a BRI. Current variable quality in discharge planning is an entry point for structural racism and disparity.

## INTRODUCTION

Bullet-related injuries (BRI) are a public health epidemic. The United States (US) averages more than 85,000 annual emergency department (ED) visits for non-fatal bullet injury.^1^ While some BRI patients will be admitted to the hospital for further management, approximately 70% are discharged from the ED to self-care and outpatient management.^1^

Patients who survive a BRI are at a vulnerable and complex moment in their lives.^2^ Many BRI patients are young, uninsured, and likely to be individuals who are otherwise healthy without regular contact with the healthcare system.^3^,^4^ This places additional burden on these patients who must manage the physical pain and emotional distress of having survived a BRI, and then learn how to care for their injury without prior wound care experience.^5^ This can create a challenging and traumatic recovery for many patients.

St. Louis, Missouri, has one of the highest rates of murder and violent crime per capita in the US, with the rate of violent firearm deaths over 14 times the national average in 2016.^6^,^7^ Our institution cares for over 600 patients with BRI per year.^3^ Our prior work found that 26% of patients discharged from our ED return within 12 months with a chief complaint most frequently related to pain or wound concerns.^8^ Given this high return rate, we investigated the anticipatory guidance and clinical resources that BRI patients receive on discharge from our institution.

Our primary objective in this study was to analyze the wound care instructions and pain management plans provided to BRI patients on discharge from the ED. We also characterize the follow-up plans and resources given to BRI patients upon their discharge from the ED. Racial minorities frequently experience health disparities in our region, and our preliminary data indicate that 86% of individuals injured by firearms in our region are Black.^9^ For this reason, we also performed a preliminary investigation of differences in care based on patient race.

## METHODS

### Study Design

This was a single-center, cross-sectional needs assessment study of a convenience sample of the first 100 consecutive patients who presented to the ED with an acute BRI in 2020. This needs-assessment study is part of a larger ED initiative to improve trauma-informed care in the ED. Patients included in this study presented to the ED between January 1–April 19, 2020. Findings are reported in accordance with the Strengthening the Reporting of Observational Studies in Epidemiology (STROBE) guidelines.^10^ This study was approved by our institutional review board.

### Study Setting and Population

This urban, academic, Level I trauma center ED with over 90,000 annual patient visits is part of the Washington University healthcare system that serves St. Louis, MO, which has a high incidence of BRI.^3^,^7^ The care of firearm-injured patients in this ED is provided by a team of over 200 clinicians including attending physicians, residents, and advanced practice practitioners.

### Study Protocol

We identified patients by mapping *International Classification of Diseases, 10^th^ Revision*, acute firearm-injury codes to SNOMED software (SNOMED International, London, United Kingdom, https://www.snomed.org/snomed-international/who-we-are). The search was accomplished using SNOMED terms in Table 1 and all descendent terms in the SNOMED hierarchies used in clinical documentation and reporting. The resultant full list of SNOMED terms is included in Appendix A.

This was a study of consecutive, adult patients (≥18 years old) who presented to the ED for care after sustaining an acute BRI. We excluded from analysis those patient visits related to follow-up care after a prior injury. Author JMH extracted study data through query of the electronic health (EHR). The EHR patient chart was manually reviewed for patient demographic information, emergency clinician notes, any new prescriptions that were ordered on discharge, and the full set of written instructions that were printed for the patient upon discharge.

### Measurements

We collected data on the following variables: age; gender (as a biologic variable); race and ethnicity (as a marker of underlying structural inequities); insurance status; cause of injury; hospital arrival and discharge timestamps; prescriptions provided on discharge; and instructions provided to patients in their printed discharge paperwork. We recorded patients’ length of stay in the ED based on the EHR timestamp from arrival for acute BRI to timestamp for discharge. We also collected data on whether patients received written wound care discharge instructions, and whether there was clinician documentation of provision of any type of wound care supplies (gauze, bacitracin, etc.).

We queried discharge instructions for written instructions regarding types of pain management provided. This included the frequency that the discharge instructions referenced over-the-counter pain medications such as non-steroidal anti-inflammatory drugs (NSAID) (ie, ibuprofen, naproxen), acetaminophen, topical therapies (ie, lidocaine patches), and non-pharmacologic options (ie, ice therapy, heat therapy, elevation, etc). We documented any new prescriptions that patients received for pain management, such as NSAIDs, acetaminophen, muscle relaxants, topical therapies, and opioids. We examined the type of opioid prescriptions and tablet counts that were prescribed and calculated the prevalence of receiving an opioid prescription at discharge based on patient race.

Query of written, follow-up instructions included analysis of outpatient follow-up care locations provided to patients in writing at discharge. This included instructions to follow up with an established primary care doctor, establish care with a new primary care doctor, follow up with a general surgery wound care clinic, or follow up with a subspecialty surgical clinic.

### Data Analysis

We summarized demographic and clinical characteristics using descriptive statistics in the form of median (interquartile range [IQR]) for continuous variables and frequency (percentage) for categorical variables. Chi-square tests were used to compare the number of opioid prescriptions provided by patient race. We conducted all analyses using Stata statistical software version 16 (StataCorp, LLC, College Station, TX).

## RESULTS

There were 100 patients who presented to the ED with an acute BRI between January 1–April 29, 2020. Patients were predominantly young (median age 29 years old, IQR 23–38), male (86%), Black (85%), non-Hispanic (98%), and uninsured (70%). The primary attributed cause of the BRI was interpersonal violence (83%). Patient demographics are detailed in Table 2. Patients spent a mean of five hours (SD 3.51) in the ED from time of arrival to time of discharge. We found that 12% of patients did not receive any type of written wound care instructions. There was not any significant racial variation in the prevalence of instruction. Only two patients were documented as having received any type of wound care supplies to take home to help with the initial dressing changes.

Additionally, we found that 71% of patients received instructions to take an NSAID and 41% received instructions to take acetaminophen, while 37% of patients received instructions to take both an NSAID and acetaminophen. Comprehensive description of pain medication, written instructions, and counts of new pain medications prescribed at time of discharge are detailed in Table 2. There was variability among opioid prescription rates. The median number of opioid tablets prescribed at discharge was 10 tablets, which ranged from 3–42 tablets. The proportion of patients receiving an opioid prescription was significantly higher (*P***=**0.05) among White patients (77%) than among Black patients (47%).

Regarding follow-up plans, 13% of patients did not receive any type of written instructions for a follow-up location. There was not any significant racial variation in the prevalence of instruction to seek follow-up care. Some patients were instructed to follow up at either a surgical wound care (34%) or subspecialty clinic (34%). While some patients (28%) were instructed to follow up with a primary care physician, only four of these patients (14%) had a previously established primary care physician.

## DISCUSSION

The results of this study reveal that our institution provides variable discharge instructions and resources to BRI survivors upon ED discharge. This included inconsistencies in both the presence and quality of written wound care instructions, as well as in whether pain medications were recommended, explained, or prescribed. As our institution does not have a standard discharge pathway, it is possible that patients received inconsistent instructions based on clinicians’ preferences, style, and oversight.

We found substantial variability in the type and quantity of pain medications prescribed on discharge. The median number of opioid tablets prescribed at discharge was 10 tablets, which ranged from 3–42 tablets. We also noted racial disparity in the proportion of patients receiving an opioid prescription (77% in White patients vs 47% in Black patients). Our findings support prior work that found non-Hispanic Blacks were less likely to receive an opioid prescription for back and abdominal pain upon ED discharge.^11^ Other studies have found that Black children are less likely than other racial groups to receive an opioid medication in the ED for conditions ranging from bone fractures to appendicitis.^12^,^13^

It is imperative for EDs to provide appropriate resources and anticipatory guidance to BRI survivors at the time of discharge. In response to these findings, we are in the process of developing a discharge smart-set in the EHR to improve standardization of discharge care in BRI survivors. Every patient who survives an acute BRI should at the very least have clear instructions about their wound care and a realistic plan for follow-up. We recognize that as we create new protocols with the aim to standardize and improve care, we must be mindful that there is appropriate flexibility, so that every patient receives patient-centered care that takes into consideration their unique circumstances.

It is our hope that these changes will improve rates at which BRI patients are provided with high-quality and appropriate instructions and resources to care for their injury. This will facilitate handoff of follow-up care to outpatient clinicians and support continued strengthening of partnerships between the ED and institutional clinics, as well as community organizations that provide BRI care.

## LIMITATIONS

This cross-sectional, needs-assessment study was designed to provide only a snapshot of what is occurring in our ED to guide future research and policy efforts to improve the delivery of trauma-informed care to our patients. There are several limitations to this study including its retrospective nature, small cohort size, and single-center design. Upcoming studies will address these limitations to improve generalizability and external validity. Additionally, there is the possibility that clinicians gave verbal pain management and wound care instructions that were not documented in the written instructions. However, this would not explain inconsistencies noted in discharge prescribing practices. Finally, we did not review patients’ history of opioid use nor differences in injury patterns, both of which may have influenced prescribing practices for pain control.

## CONCLUSION

There is notable variability in the prescriptions and instructions provided to survivors of bullet-related injuries upon discharge from the ED. The data indicates the need for standardization of practice to ensure that BRI patients are provided with equitable and consistent care.

## Supplementary Information



## Figures and Tables

**Table 1:** Terms for bullet-related injuries from the SNOMED collection of clinical terms.

eSNOMED ID	SNOMED Term
219335006	Handgun
219337003	Hunting rifle
219338008	Military firearm
219336007	Shotgun
219257002	Legal intervention
283545005	GSW
243000002	Injury due to bullet

*GSW*, gunshot wound.

**Table 2:** Demographics, injury characteristics, and discharge pain medications provided to 100 patients discharged from the emergency department after bullet-related injury.

	Count & %
Age in years (Median, IQR)	29 (23–38)
Race	
Black	85
White	13
Asian	1
Not specified	1
Gender	
Male	86
Female	14
Cause of injury	
Interpersonal violence	83
Accidental	14
Self-inflicted	1
Not specified	2
Insurance	
Self-pay	70
Private	15
Medicare	1
Medicaid	14
Instructions referenced*	
Acetaminophen	41
NSAIDs	71
Muscle relaxants	0
Topical therapies	3
Opioids	49
Non-pharmacologic therapies	59
Prescribed at discharge**	
Acetaminophen	25
NSAIDs	39
Muscle relaxants	0
Topical therapies	2
Gabapentin	2
Opioids	51
Morphine 15 mg	8 (16%)
Morphine 30 mg	4 (8%)
Oxycodone 5 mg	14 (27%)
Hydrocodone-acetaminophen 5–325 mg	20 (39%)
Oxycodone-acetaminophen 5–325 mg	4 (8%)
Tramadol 50 mg	1 (2%)

* Some patients received instructions about more than one medication.

** Some patients received a prescription for more than one medication.

*IQR*, interquartile range; *NSAIDs*, non-steroidal anti-inflammatory drugs; *mg*, milligram.
