# Supplementary material for: Care of Bullet-related Injuries: A Cross-sectional Study of Instructions and Prescriptions Provided on Discharge from the Emergency Department
Source: West J Emerg Med. 2023 Feb 27;24(2):363–7. doi: 10.5811/westjem.2022.11.57574 (PMC10047738; doi:10.5811/westjem.2022.11.57574)
Supplement: Supplementary file 1 [file wjem-24-363-s001.docx]

**Appendix A.** Full list of SNOMED TERMS

| Fracture of thumb due to gunshot wound (disorder) | SNOMED#16000001000004109 |
| --- | --- |
| Fracture of thumb due to gunshot wound (disorder) | SNOMED#16000001000004109 |
| Fracture of thumb due to gunshot wound (disorder) | SNOMED#16000001000004109 |
| Fracture of thumb due to gunshot wound (disorder) | SNOMED#16000001000004109 |
| Fracture of thumb due to gunshot wound (disorder) | SNOMED#16000001000004109 |
| Fracture of thumb due to gunshot wound (disorder) | SNOMED#16000001000004109 |
| Fracture of thumb due to gunshot wound (disorder) | SNOMED#16000001000004109 |
| Fracture of thumb due to gunshot wound (disorder) | SNOMED#16000001000004109 |
| Fracture of thumb due to gunshot wound (disorder) | SNOMED#16000001000004109 |
| Fracture of thumb due to gunshot wound (disorder) | SNOMED#16000001000004109 |
| Fracture of thumb due to gunshot wound (disorder) | SNOMED#16000001000004109 |
| Fracture of thumb due to gunshot wound (disorder) | SNOMED#16000001000004109 |
| Fracture of thumb due to gunshot wound (disorder) | SNOMED#16000001000004109 |
| Fracture of thumb due to gunshot wound (disorder) | SNOMED#16000001000004109 |
| Fracture of thumb due to gunshot wound (disorder) | SNOMED#16000001000004109 |
| Fracture of thumb due to gunshot wound (disorder) | SNOMED#16000001000004109 |
| Fracture of thumb due to gunshot wound (disorder) | SNOMED#16000001000004109 |
| Fracture of thumb due to gunshot wound (disorder) | SNOMED#16000001000004109 |
| Fracture of thumb due to gunshot wound (disorder) | SNOMED#16000001000004109 |
| Fracture of thumb due to gunshot wound (disorder) | SNOMED#16000001000004109 |
| Gunshot entry wound (disorder) | SNOMED#425055008 |
| Gunshot exit wound (disorder) | SNOMED#424867003 |
| Gunshot wound (disorder) | SNOMED#283545005 |
| Gunshot wound (disorder) | SNOMED#283545005 |
| Gunshot wound (disorder) | SNOMED#283545005 |
| Gunshot wound (disorder) | SNOMED#283545005 |
| Gunshot wound (disorder) | SNOMED#283545005 |
| Gunshot wound (disorder) | SNOMED#283545005 |
| Gunshot wound (disorder) | SNOMED#283545005 |
| Gunshot wound (disorder) | SNOMED#283545005 |
| Gunshot wound (disorder) | SNOMED#283545005 |
| Gunshot wound (disorder) | SNOMED#283545005 |
| Gunshot wound (disorder) | SNOMED#283545005 |
| Gunshot wound (disorder) | SNOMED#283545005 |
| Gunshot wound (disorder) | SNOMED#283545005 |
| Gunshot wound (disorder) | SNOMED#283545005 |
| Gunshot wound (disorder) | SNOMED#283545005 |
| Gunshot wound (disorder) | SNOMED#283545005 |
| Gunshot wound (disorder) | SNOMED#283545005 |
| Gunshot wound (disorder) | SNOMED#283545005 |
| Gunshot wound (disorder) | SNOMED#283545005 |
| Gunshot wound (disorder) | SNOMED#283545005 |
| Gunshot wound (disorder) | SNOMED#283545005 |
| Gunshot wound (disorder) | SNOMED#283545005 |
| Gunshot wound (disorder) | SNOMED#283545005 |
| Gunshot wound (disorder) | SNOMED#283545005 |
| Gunshot wound (disorder) | SNOMED#283545005 |
| Gunshot wound (disorder) | SNOMED#283545005 |
| Gunshot wound (disorder) | SNOMED#283545005 |
| Gunshot wound (disorder) | SNOMED#283545005 |
| Gunshot wound (disorder) | SNOMED#283545005 |
| Gunshot wound (disorder) | SNOMED#283545005 |
| Gunshot wound (disorder) | SNOMED#283545005 |
| Gunshot wound (disorder) | SNOMED#283545005 |
| Gunshot wound (disorder) | SNOMED#283545005 |
| Gunshot wound (disorder) | SNOMED#283545005 |
| Gunshot wound (disorder) | SNOMED#283545005 |
| Gunshot wound (disorder) | SNOMED#283545005 |
| Gunshot wound (disorder) | SNOMED#283545005 |
| Gunshot wound (disorder) | SNOMED#283545005 |
| Gunshot wound (disorder) | SNOMED#283545005 |
| Gunshot wound (disorder) | SNOMED#283545005 |
| Gunshot wound (disorder) | SNOMED#283545005 |
| Gunshot wound (disorder) | SNOMED#283545005 |
| Gunshot wound (disorder) | SNOMED#283545005 |
| Gunshot wound (disorder) | SNOMED#283545005 |
| Gunshot wound (disorder) | SNOMED#283545005 |
| Gunshot wound (disorder) | SNOMED#283545005 |
| Gunshot wound (disorder) | SNOMED#283545005 |
| Gunshot wound (disorder) | SNOMED#283545005 |
| Gunshot wound (disorder) | SNOMED#283545005 |
| Gunshot wound (disorder) | SNOMED#283545005 |
| Gunshot wound (disorder) | SNOMED#283545005 |
| Gunshot wound (disorder) | SNOMED#283545005 |
| Gunshot wound (disorder) | SNOMED#283545005 |
| Gunshot wound (disorder) | SNOMED#283545005 |
| Gunshot wound (disorder) | SNOMED#283545005 |
| Gunshot wound (disorder) | SNOMED#283545005 |
| Gunshot wound (disorder) | SNOMED#283545005 |
| Gunshot wound (disorder) | SNOMED#283545005 |
| Gunshot wound (disorder) | SNOMED#283545005 |
| Gunshot wound (disorder) | SNOMED#283545005 |
| Gunshot wound (disorder) | SNOMED#283545005 |
| Gunshot wound (disorder) | SNOMED#283545005 |
| Gunshot wound (disorder) | SNOMED#283545005 |
| Gunshot wound (disorder) | SNOMED#283545005 |
| Gunshot wound (disorder) | SNOMED#283545005 |
| Gunshot wound (disorder) | SNOMED#283545005 |
| Gunshot wound (disorder) | SNOMED#283545005 |
| Gunshot wound (disorder) | SNOMED#283545005 |
| Gunshot wound (disorder) | SNOMED#283545005 |
| Gunshot wound (disorder) | SNOMED#283545005 |
| Gunshot wound (disorder) | SNOMED#283545005 |
| Gunshot wound (disorder) | SNOMED#283545005 |
| Gunshot wound (disorder) | SNOMED#283545005 |
| Gunshot wound (disorder) | SNOMED#283545005 |
| Gunshot wound (disorder) | SNOMED#283545005 |
| Gunshot wound (disorder) | SNOMED#283545005 |
| Gunshot wound (disorder) | SNOMED#283545005 |
| Gunshot wound (disorder) | SNOMED#283545005 |
| Gunshot wound (disorder) | SNOMED#283545005 |
| Gunshot wound (disorder) | SNOMED#283545005 |
| Gunshot wound (disorder) | SNOMED#283545005 |
| Gunshot wound (disorder) | SNOMED#283545005 |
| Gunshot wound (disorder) | SNOMED#283545005 |
| Gunshot wound (disorder) | SNOMED#283545005 |
| Gunshot wound (disorder) | SNOMED#283545005 |
| Gunshot wound (disorder) | SNOMED#283545005 |
| Gunshot wound (disorder) | SNOMED#283545005 |
| Gunshot wound (disorder) | SNOMED#283545005 |
| Gunshot wound (disorder) | SNOMED#283545005 |
| Gunshot wound (disorder) | SNOMED#283545005 |
| Gunshot wound (disorder) | SNOMED#283545005 |
| Gunshot wound (disorder) | SNOMED#283545005 |
| Gunshot wound (disorder) | SNOMED#283545005 |
| Gunshot wound (disorder) | SNOMED#283545005 |
| Gunshot wound (disorder) | SNOMED#283545005 |
| Gunshot wound (disorder) | SNOMED#283545005 |
| Gunshot wound (disorder) | SNOMED#283545005 |
| Gunshot wound (disorder) | SNOMED#283545005 |
| Gunshot wound (disorder) | SNOMED#283545005 |
| Gunshot wound (disorder) | SNOMED#283545005 |
| Gunshot wound (disorder) | SNOMED#283545005 |
| Gunshot wound (disorder) | SNOMED#283545005 |
| Gunshot wound (disorder) | SNOMED#283545005 |
| Gunshot wound (disorder) | SNOMED#283545005 |
| Gunshot wound (disorder) | SNOMED#283545005 |
| Gunshot wound (disorder) | SNOMED#283545005 |
| Gunshot wound (disorder) | SNOMED#283545005 |
| Gunshot wound (disorder) | SNOMED#283545005 |
| Gunshot wound (disorder) | SNOMED#283545005 |
| Gunshot wound (disorder) | SNOMED#283545005 |
| Gunshot wound (disorder) | SNOMED#283545005 |
| Gunshot wound (disorder) | SNOMED#283545005 |
| Gunshot wound (disorder) | SNOMED#283545005 |
| Gunshot wound (disorder) | SNOMED#283545005 |
| Gunshot wound (disorder) | SNOMED#283545005 |
| Gunshot wound (disorder) | SNOMED#283545005 |
| Gunshot wound (disorder) | SNOMED#283545005 |
| Gunshot wound (disorder) | SNOMED#283545005 |
| Gunshot wound (disorder) | SNOMED#283545005 |
| Gunshot wound (disorder) | SNOMED#283545005 |
| Gunshot wound (disorder) | SNOMED#283545005 |
| Gunshot wound (disorder) | SNOMED#283545005 |
| Gunshot wound (disorder) | SNOMED#283545005 |
| Gunshot wound (disorder) | SNOMED#283545005 |
| Gunshot wound (disorder) | SNOMED#283545005 |
| Gunshot wound (disorder) | SNOMED#283545005 |
| Gunshot wound (disorder) | SNOMED#283545005 |
| Gunshot wound (disorder) | SNOMED#283545005 |
| Gunshot wound (disorder) | SNOMED#283545005 |
| Gunshot wound (disorder) | SNOMED#283545005 |
| Gunshot wound (disorder) | SNOMED#283545005 |
| Gunshot wound (disorder) | SNOMED#283545005 |
| Gunshot wound (disorder) | SNOMED#283545005 |
| Gunshot wound (disorder) | SNOMED#283545005 |
| Gunshot wound (disorder) | SNOMED#283545005 |
| Gunshot wound (disorder) | SNOMED#283545005 |
| Gunshot wound (disorder) | SNOMED#283545005 |
| Gunshot wound (disorder) | SNOMED#283545005 |
| Gunshot wound (disorder) | SNOMED#283545005 |
| Gunshot wound (disorder) | SNOMED#283545005 |
| Gunshot wound (disorder) | SNOMED#283545005 |
| Gunshot wound (disorder) | SNOMED#283545005 |
| Gunshot wound (disorder) | SNOMED#283545005 |
| Gunshot wound (disorder) | SNOMED#283545005 |
| Gunshot wound (disorder) | SNOMED#283545005 |
| Gunshot wound (disorder) | SNOMED#283545005 |
| Gunshot wound (disorder) | SNOMED#283545005 |
| Gunshot wound (disorder) | SNOMED#283545005 |
| Gunshot wound (disorder) | SNOMED#283545005 |
| Gunshot wound (disorder) | SNOMED#283545005 |
| Gunshot wound (disorder) | SNOMED#283545005 |
| Gunshot wound (disorder) | SNOMED#283545005 |
| Gunshot wound (disorder) | SNOMED#283545005 |
| Gunshot wound (disorder) | SNOMED#283545005 |
| Gunshot wound (disorder) | SNOMED#283545005 |
| Gunshot wound (disorder) | SNOMED#283545005 |
| Gunshot wound (disorder) | SNOMED#283545005 |
| Gunshot wound (disorder) | SNOMED#283545005 |
| Gunshot wound (disorder) | SNOMED#283545005 |
| Gunshot wound (disorder) | SNOMED#283545005 |
| Gunshot wound (disorder) | SNOMED#283545005 |
| Gunshot wound (disorder) | SNOMED#283545005 |
| Gunshot wound (disorder) | SNOMED#283545005 |
| Gunshot wound (disorder) | SNOMED#283545005 |
| Gunshot wound (disorder) | SNOMED#283545005 |
| Gunshot wound (disorder) | SNOMED#283545005 |
| Gunshot wound (disorder) | SNOMED#283545005 |
| Gunshot wound (disorder) | SNOMED#283545005 |
| Gunshot wound (disorder) | SNOMED#283545005 |
| Gunshot wound (disorder) | SNOMED#283545005 |
| Gunshot wound (disorder) | SNOMED#283545005 |
| Gunshot wound (disorder) | SNOMED#283545005 |
| Gunshot wound (disorder) | SNOMED#283545005 |
| Gunshot wound (disorder) | SNOMED#283545005 |
| Gunshot wound (disorder) | SNOMED#283545005 |
| Gunshot wound (disorder) | SNOMED#283545005 |
| Gunshot wound (disorder) | SNOMED#283545005 |
| Gunshot wound (disorder) | SNOMED#283545005 |
| Gunshot wound (disorder) | SNOMED#283545005 |
| Gunshot wound (disorder) | SNOMED#283545005 |
| Gunshot wound (disorder) | SNOMED#283545005 |
| Gunshot wound (disorder) | SNOMED#283545005 |
| Gunshot wound (disorder) | SNOMED#283545005 |
| Gunshot wound (disorder) | SNOMED#283545005 |
| Gunshot wound (disorder) | SNOMED#283545005 |
| Gunshot wound (disorder) | SNOMED#283545005 |
| Gunshot wound (disorder) | SNOMED#283545005 |
| Gunshot wound (disorder) | SNOMED#283545005 |
| Gunshot wound (disorder) | SNOMED#283545005 |
| Gunshot wound (disorder) | SNOMED#283545005 |
| Gunshot wound (disorder) | SNOMED#283545005 |
| Gunshot wound (disorder) | SNOMED#283545005 |
| Gunshot wound (disorder) | SNOMED#283545005 |
| Gunshot wound (disorder) | SNOMED#283545005 |
| Gunshot wound (disorder) | SNOMED#283545005 |
| Gunshot wound (disorder) | SNOMED#283545005 |
| Gunshot wound (disorder) | SNOMED#283545005 |
| Gunshot wound (disorder) | SNOMED#283545005 |
| Gunshot wound (disorder) | SNOMED#283545005 |
| Gunshot wound (disorder) | SNOMED#283545005 |
| Gunshot wound (disorder) | SNOMED#283545005 |
| Gunshot wound (disorder) | SNOMED#283545005 |
| Gunshot wound (disorder) | SNOMED#283545005 |
| Gunshot wound (disorder) | SNOMED#283545005 |
| Gunshot wound (disorder) | SNOMED#283545005 |
| Gunshot wound (disorder) | SNOMED#283545005 |
| Gunshot wound (disorder) | SNOMED#283545005 |
| Gunshot wound (disorder) | SNOMED#283545005 |
| Gunshot wound (disorder) | SNOMED#283545005 |
| Gunshot wound (disorder) | SNOMED#283545005 |
| Gunshot wound (disorder) | SNOMED#283545005 |
| Gunshot wound (disorder) | SNOMED#283545005 |
| Gunshot wound (disorder) | SNOMED#283545005 |
| Gunshot wound (disorder) | SNOMED#283545005 |
| Gunshot wound (disorder) | SNOMED#283545005 |
| Gunshot wound (disorder) | SNOMED#283545005 |
| Gunshot wound (disorder) | SNOMED#283545005 |
| Gunshot wound (disorder) | SNOMED#283545005 |
| Gunshot wound (disorder) | SNOMED#283545005 |
| Gunshot wound (disorder) | SNOMED#283545005 |
| Gunshot wound (disorder) | SNOMED#283545005 |
| Gunshot wound (disorder) | SNOMED#283545005 |
| Gunshot wound (disorder) | SNOMED#283545005 |
| Gunshot wound (disorder) | SNOMED#283545005 |
| Gunshot wound (disorder) | SNOMED#283545005 |
| Gunshot wound (disorder) | SNOMED#283545005 |
| Gunshot wound (disorder) | SNOMED#283545005 |
| Gunshot wound (disorder) | SNOMED#283545005 |
| Gunshot wound (disorder) | SNOMED#283545005 |
| Gunshot wound (disorder) | SNOMED#283545005 |
| Gunshot wound (disorder) | SNOMED#283545005 |
| Gunshot wound (disorder) | SNOMED#283545005 |
| Gunshot wound (disorder) | SNOMED#283545005 |
| Gunshot wound (disorder) | SNOMED#283545005 |
| Gunshot wound (disorder) | SNOMED#283545005 |
| Gunshot wound (disorder) | SNOMED#283545005 |
| Gunshot wound (disorder) | SNOMED#283545005 |
| Gunshot wound (disorder) | SNOMED#283545005 |
| Gunshot wound (disorder) | SNOMED#283545005 |
| Gunshot wound (disorder) | SNOMED#283545005 |
| Gunshot wound (disorder) | SNOMED#283545005 |
| Gunshot wound (disorder) | SNOMED#283545005 |
| Gunshot wound (disorder) | SNOMED#283545005 |
| Gunshot wound (disorder) | SNOMED#283545005 |
| Gunshot wound (disorder) | SNOMED#283545005 |
| Gunshot wound (disorder) | SNOMED#283545005 |
| Gunshot wound (disorder) | SNOMED#283545005 |
| Gunshot wound (disorder) | SNOMED#283545005 |
| Gunshot wound (disorder) | SNOMED#283545005 |
| Gunshot wound (disorder) | SNOMED#283545005 |
| Gunshot wound (disorder) | SNOMED#283545005 |
| Gunshot wound (disorder) | SNOMED#283545005 |
| Gunshot wound (disorder) | SNOMED#283545005 |
| Gunshot wound (disorder) | SNOMED#283545005 |
| Gunshot wound (disorder) | SNOMED#283545005 |
| Gunshot wound (disorder) | SNOMED#283545005 |
| Gunshot wound (disorder) | SNOMED#283545005 |
| Gunshot wound (disorder) | SNOMED#283545005 |
| Gunshot wound (disorder) | SNOMED#283545005 |
| Gunshot wound (disorder) | SNOMED#283545005 |
| Gunshot wound (disorder) | SNOMED#283545005 |
| Gunshot wound (disorder) | SNOMED#283545005 |
| Gunshot wound (disorder) | SNOMED#283545005 |
| Gunshot wound (disorder) | SNOMED#283545005 |
| Gunshot wound (disorder) | SNOMED#283545005 |
| Gunshot wound (disorder) | SNOMED#283545005 |
| Gunshot wound (disorder) | SNOMED#283545005 |
| Gunshot wound (disorder) | SNOMED#283545005 |
| Gunshot wound (disorder) | SNOMED#283545005 |
| Gunshot wound (disorder) | SNOMED#283545005 |
| Gunshot wound (disorder) | SNOMED#283545005 |
| Gunshot wound (disorder) | SNOMED#283545005 |
| Gunshot wound (disorder) | SNOMED#283545005 |
| Gunshot wound (disorder) | SNOMED#283545005 |
| Gunshot wound (disorder) | SNOMED#283545005 |
| Gunshot wound (disorder) | SNOMED#283545005 |
| Gunshot wound (disorder) | SNOMED#283545005 |
| Gunshot wound (disorder) | SNOMED#283545005 |
| Gunshot wound (disorder) | SNOMED#283545005 |
| Gunshot wound (disorder) | SNOMED#283545005 |
| Gunshot wound (disorder) | SNOMED#283545005 |
| Gunshot wound (disorder) | SNOMED#283545005 |
| Gunshot wound (disorder) | SNOMED#283545005 |
| Gunshot wound (disorder) | SNOMED#283545005 |
| Gunshot wound (disorder) | SNOMED#283545005 |
| Gunshot wound (disorder) | SNOMED#283545005 |
| Gunshot wound (disorder) | SNOMED#283545005 |
| Gunshot wound (disorder) | SNOMED#283545005 |
| Gunshot wound (disorder) | SNOMED#283545005 |
| Gunshot wound (disorder) | SNOMED#283545005 |
| Gunshot wound (disorder) | SNOMED#283545005 |
| Gunshot wound (disorder) | SNOMED#283545005 |
| Gunshot wound (disorder) | SNOMED#283545005 |
| Gunshot wound (disorder) | SNOMED#283545005 |
| Gunshot wound (disorder) | SNOMED#283545005 |
| Gunshot wound (disorder) | SNOMED#283545005 |
| Gunshot wound (disorder) | SNOMED#283545005 |
| Gunshot wound (disorder) | SNOMED#283545005 |
| Gunshot wound (disorder) | SNOMED#283545005 |
| Gunshot wound (disorder) | SNOMED#283545005 |
| Gunshot wound (disorder) | SNOMED#283545005 |
| Gunshot wound (disorder) | SNOMED#283545005 |
| Gunshot wound (disorder) | SNOMED#283545005 |
| Gunshot wound (disorder) | SNOMED#283545005 |
| Gunshot wound (disorder) | SNOMED#283545005 |
| Gunshot wound (disorder) | SNOMED#283545005 |
| Gunshot wound (disorder) | SNOMED#283545005 |
| Gunshot wound (disorder) | SNOMED#283545005 |
| Gunshot wound (disorder) | SNOMED#283545005 |
| Gunshot wound (disorder) | SNOMED#283545005 |
| Gunshot wound (disorder) | SNOMED#283545005 |
| Gunshot wound (disorder) | SNOMED#283545005 |
| Gunshot wound (disorder) | SNOMED#283545005 |
| Gunshot wound (disorder) | SNOMED#283545005 |
| Gunshot wound (disorder) | SNOMED#283545005 |
| Gunshot wound (disorder) | SNOMED#283545005 |
| Gunshot wound (disorder) | SNOMED#283545005 |
| Gunshot wound (disorder) | SNOMED#283545005 |
| Gunshot wound (disorder) | SNOMED#283545005 |
| Gunshot wound (disorder) | SNOMED#283545005 |
| Gunshot wound (disorder) | SNOMED#283545005 |
| Gunshot wound (disorder) | SNOMED#283545005 |
| Gunshot wound (disorder) | SNOMED#283545005 |
| Gunshot wound (disorder) | SNOMED#283545005 |
| Gunshot wound (disorder) | SNOMED#283545005 |
| Gunshot wound (disorder) | SNOMED#283545005 |
| Gunshot wound (disorder) | SNOMED#283545005 |
| Gunshot wound (disorder) | SNOMED#283545005 |
| Gunshot wound (disorder) | SNOMED#283545005 |
| Gunshot wound (disorder) | SNOMED#283545005 |
| Gunshot wound (disorder) | SNOMED#283545005 |
| Gunshot wound (disorder) | SNOMED#283545005 |
| Gunshot wound (disorder) | SNOMED#283545005 |
| Gunshot wound (disorder) | SNOMED#283545005 |
| Gunshot wound (disorder) | SNOMED#283545005 |
| Gunshot wound (disorder) | SNOMED#283545005 |
| Gunshot wound (disorder) | SNOMED#283545005 |
| Gunshot wound (disorder) | SNOMED#283545005 |
| Gunshot wound (disorder) | SNOMED#283545005 |
| Gunshot wound (disorder) | SNOMED#283545005 |
| Gunshot wound (disorder) | SNOMED#283545005 |
| Gunshot wound (disorder) | SNOMED#283545005 |
| Gunshot wound (disorder) | SNOMED#283545005 |
| Gunshot wound (disorder) | SNOMED#283545005 |
| Gunshot wound (disorder) | SNOMED#283545005 |
| Gunshot wound (disorder) | SNOMED#283545005 |
| Gunshot wound (disorder) | SNOMED#283545005 |
| Gunshot wound of abdomen region (disorder) | SNOMED#23071000175104 |
| Gunshot wound of abdomen region (disorder) | SNOMED#23071000175104 |
| Gunshot wound of abdomen region (disorder) | SNOMED#23071000175104 |
| Gunshot wound of abdominal wall (disorder) | SNOMED#10878111000119106 |
| Gunshot wound of abdominal wall (disorder) | SNOMED#10878111000119106 |
| Gunshot wound of abdominal wall (disorder) | SNOMED#10878111000119106 |
| Gunshot wound of chest (disorder) | SNOMED#23061000175107 |
| Gunshot wound of chest (disorder) | SNOMED#23061000175107 |
| Gunshot wound of chest (disorder) | SNOMED#23061000175107 |
| Gunshot wound of extremity (disorder) | SNOMED#23081000175101 |
| Gunshot wound of face (disorder) | SNOMED#10907361000119107 |
| Gunshot wound of face (disorder) | SNOMED#10907361000119107 |
| Gunshot wound of face (disorder) | SNOMED#10907361000119107 |
| Gunshot wound of face (disorder) | SNOMED#10907361000119107 |
| Gunshot wound of face (disorder) | SNOMED#10907361000119107 |
| Gunshot wound of face (disorder) | SNOMED#10907361000119107 |
| Gunshot wound of face (disorder) | SNOMED#10907361000119107 |
| Gunshot wound of face (disorder) | SNOMED#10907361000119107 |
| Gunshot wound of face (disorder) | SNOMED#10907361000119107 |
| Gunshot wound of face (disorder) | SNOMED#10907361000119107 |
| Gunshot wound of face (disorder) | SNOMED#10907361000119107 |
| Gunshot wound of face (disorder) | SNOMED#10907361000119107 |
| Gunshot wound of face (disorder) | SNOMED#10907361000119107 |
| Gunshot wound of face (disorder) | SNOMED#10907361000119107 |
| Gunshot wound of face (disorder) | SNOMED#10907361000119107 |
| Gunshot wound of foot (disorder) | SNOMED#23121000175104 |
| Gunshot wound of foot (disorder) | SNOMED#23121000175104 |
| Gunshot wound of foot (disorder) | SNOMED#23121000175104 |
| Gunshot wound of hand (disorder) | SNOMED#23101000175105 |
| Gunshot wound of hand (disorder) | SNOMED#23101000175105 |
| Gunshot wound of hand (disorder) | SNOMED#23101000175105 |
| Gunshot wound of head (disorder) | SNOMED#23051000175105 |
| Gunshot wound of head (disorder) | SNOMED#23051000175105 |
| Gunshot wound of head (disorder) | SNOMED#23051000175105 |
| Gunshot wound of left ankle region (disorder) | SNOMED#10863881000119106 |
| Gunshot wound of left ankle region (disorder) | SNOMED#10863881000119106 |
| Gunshot wound of left ankle region (disorder) | SNOMED#10863881000119106 |
| Gunshot wound of left ankle region (disorder) | SNOMED#10863881000119106 |
| Gunshot wound of left ankle region (disorder) | SNOMED#10863881000119106 |
| Gunshot wound of left ankle region (disorder) | SNOMED#10863881000119106 |
| Gunshot wound of left axillary region (disorder) | SNOMED#10907561000119101 |
| Gunshot wound of left axillary region (disorder) | SNOMED#10907561000119101 |
| Gunshot wound of left axillary region (disorder) | SNOMED#10907561000119101 |
| Gunshot wound of left axillary region (disorder) | SNOMED#10907561000119101 |
| Gunshot wound of left axillary region (disorder) | SNOMED#10907561000119101 |
| Gunshot wound of left axillary region (disorder) | SNOMED#10907561000119101 |
| Gunshot wound of left buttock (disorder) | SNOMED#10907721000119103 |
| Gunshot wound of left buttock (disorder) | SNOMED#10907721000119103 |
| Gunshot wound of left buttock (disorder) | SNOMED#10907721000119103 |
| Gunshot wound of left foot (disorder) | SNOMED#10863981000119100 |
| Gunshot wound of left foot (disorder) | SNOMED#10863981000119100 |
| Gunshot wound of left foot (disorder) | SNOMED#10863981000119100 |
| Gunshot wound of left foot (disorder) | SNOMED#10863981000119100 |
| Gunshot wound of left foot (disorder) | SNOMED#10863981000119100 |
| Gunshot wound of left foot (disorder) | SNOMED#10863981000119100 |
| Gunshot wound of left foot (disorder) | SNOMED#10863981000119100 |
| Gunshot wound of left foot (disorder) | SNOMED#10863981000119100 |
| Gunshot wound of left foot (disorder) | SNOMED#10863981000119100 |
| Gunshot wound of left foot (disorder) | SNOMED#10863981000119100 |
| Gunshot wound of left foot (disorder) | SNOMED#10863981000119100 |
| Gunshot wound of left foot (disorder) | SNOMED#10863981000119100 |
| Gunshot wound of left foot (disorder) | SNOMED#10863981000119100 |
| Gunshot wound of left foot (disorder) | SNOMED#10863981000119100 |
| Gunshot wound of left foot (disorder) | SNOMED#10863981000119100 |
| Gunshot wound of left forearm (disorder) | SNOMED#10950111000119103 |
| Gunshot wound of left forearm (disorder) | SNOMED#10950111000119103 |
| Gunshot wound of left forearm (disorder) | SNOMED#10950111000119103 |
| Gunshot wound of left forearm (disorder) | SNOMED#10950111000119103 |
| Gunshot wound of left forearm (disorder) | SNOMED#10950111000119103 |
| Gunshot wound of left forearm (disorder) | SNOMED#10950111000119103 |
| Gunshot wound of left hand (disorder) | SNOMED#10950231000119103 |
| Gunshot wound of left hand (disorder) | SNOMED#10950231000119103 |
| Gunshot wound of left hand (disorder) | SNOMED#10950231000119103 |
| Gunshot wound of left hand (disorder) | SNOMED#10950231000119103 |
| Gunshot wound of left hand (disorder) | SNOMED#10950231000119103 |
| Gunshot wound of left hand (disorder) | SNOMED#10950231000119103 |
| Gunshot wound of left hand (disorder) | SNOMED#10950231000119103 |
| Gunshot wound of left hand (disorder) | SNOMED#10950231000119103 |
| Gunshot wound of left hand (disorder) | SNOMED#10950231000119103 |
| Gunshot wound of left hand (disorder) | SNOMED#10950231000119103 |
| Gunshot wound of left hand (disorder) | SNOMED#10950231000119103 |
| Gunshot wound of left hand (disorder) | SNOMED#10950231000119103 |
| Gunshot wound of left hand (disorder) | SNOMED#10950231000119103 |
| Gunshot wound of left hand (disorder) | SNOMED#10950231000119103 |
| Gunshot wound of left hand (disorder) | SNOMED#10950231000119103 |
| Gunshot wound of left hand (disorder) | SNOMED#10950231000119103 |
| Gunshot wound of left hand (disorder) | SNOMED#10950231000119103 |
| Gunshot wound of left hand (disorder) | SNOMED#10950231000119103 |
| Gunshot wound of left hand (disorder) | SNOMED#10950231000119103 |
| Gunshot wound of left hand (disorder) | SNOMED#10950231000119103 |
| Gunshot wound of left hip region (disorder) | SNOMED#10861691000119106 |
| Gunshot wound of left hip region (disorder) | SNOMED#10861691000119106 |
| Gunshot wound of left hip region (disorder) | SNOMED#10861691000119106 |
| Gunshot wound of left hip region (disorder) | SNOMED#10861691000119106 |
| Gunshot wound of left hip region (disorder) | SNOMED#10861691000119106 |
| Gunshot wound of left hip region (disorder) | SNOMED#10861691000119106 |
| Gunshot wound of left knee region (disorder) | SNOMED#10862131000119109 |
| Gunshot wound of left knee region (disorder) | SNOMED#10862131000119109 |
| Gunshot wound of left knee region (disorder) | SNOMED#10862131000119109 |
| Gunshot wound of left knee region (disorder) | SNOMED#10862131000119109 |
| Gunshot wound of left knee region (disorder) | SNOMED#10862131000119109 |
| Gunshot wound of left knee region (disorder) | SNOMED#10862131000119109 |
| Gunshot wound of left lower leg (disorder) | SNOMED#10864111000119107 |
| Gunshot wound of left lower leg (disorder) | SNOMED#10864111000119107 |
| Gunshot wound of left lower leg (disorder) | SNOMED#10864111000119107 |
| Gunshot wound of left lower leg (disorder) | SNOMED#10864111000119107 |
| Gunshot wound of left lower leg (disorder) | SNOMED#10864111000119107 |
| Gunshot wound of left lower leg (disorder) | SNOMED#10864111000119107 |
| Gunshot wound of left shoulder region (disorder) | SNOMED#10878231000119106 |
| Gunshot wound of left shoulder region (disorder) | SNOMED#10878231000119106 |
| Gunshot wound of left shoulder region (disorder) | SNOMED#10878231000119106 |
| Gunshot wound of left shoulder region (disorder) | SNOMED#10878231000119106 |
| Gunshot wound of left shoulder region (disorder) | SNOMED#10878231000119106 |
| Gunshot wound of left shoulder region (disorder) | SNOMED#10878231000119106 |
| Gunshot wound of left thigh (disorder) | SNOMED#10861931000119102 |
| Gunshot wound of left thigh (disorder) | SNOMED#10861931000119102 |
| Gunshot wound of left thigh (disorder) | SNOMED#10861931000119102 |
| Gunshot wound of left thigh (disorder) | SNOMED#10861931000119102 |
| Gunshot wound of left thigh (disorder) | SNOMED#10861931000119102 |
| Gunshot wound of left thigh (disorder) | SNOMED#10861931000119102 |
| Gunshot wound of left upper arm (disorder) | SNOMED#10907521000119106 |
| Gunshot wound of left upper arm (disorder) | SNOMED#10907521000119106 |
| Gunshot wound of left upper arm (disorder) | SNOMED#10907521000119106 |
| Gunshot wound of left upper arm (disorder) | SNOMED#10907521000119106 |
| Gunshot wound of left upper arm (disorder) | SNOMED#10907521000119106 |
| Gunshot wound of left upper arm (disorder) | SNOMED#10907521000119106 |
| Gunshot wound of lower back (disorder) | SNOMED#10866851000119108 |
| Gunshot wound of lower limb (disorder) | SNOMED#23111000175108 |
| Gunshot wound of lower limb (disorder) | SNOMED#23111000175108 |
| Gunshot wound of lower limb (disorder) | SNOMED#23111000175108 |
| Gunshot wound of neck (disorder) | SNOMED#10954491000119108 |
| Gunshot wound of neck (disorder) | SNOMED#10954491000119108 |
| Gunshot wound of neck (disorder) | SNOMED#10954491000119108 |
| Gunshot wound of neck (disorder) | SNOMED#10954491000119108 |
| Gunshot wound of neck (disorder) | SNOMED#10954491000119108 |
| Gunshot wound of neck (disorder) | SNOMED#10954491000119108 |
| Gunshot wound of pelvis (disorder) | SNOMED#10866811000119107 |
| Gunshot wound of pelvis (disorder) | SNOMED#10866811000119107 |
| Gunshot wound of pelvis (disorder) | SNOMED#10866811000119107 |
| Gunshot wound of right ankle region (disorder) | SNOMED#10863911000119106 |
| Gunshot wound of right ankle region (disorder) | SNOMED#10863911000119106 |
| Gunshot wound of right ankle region (disorder) | SNOMED#10863911000119106 |
| Gunshot wound of right ankle region (disorder) | SNOMED#10863911000119106 |
| Gunshot wound of right ankle region (disorder) | SNOMED#10863911000119106 |
| Gunshot wound of right ankle region (disorder) | SNOMED#10863911000119106 |
| Gunshot wound of right axillary region (disorder) | SNOMED#10907481000119106 |
| Gunshot wound of right axillary region (disorder) | SNOMED#10907481000119106 |
| Gunshot wound of right axillary region (disorder) | SNOMED#10907481000119106 |
| Gunshot wound of right axillary region (disorder) | SNOMED#10907481000119106 |
| Gunshot wound of right axillary region (disorder) | SNOMED#10907481000119106 |
| Gunshot wound of right axillary region (disorder) | SNOMED#10907481000119106 |
| Gunshot wound of right buttock (disorder) | SNOMED#10878191000119102 |
| Gunshot wound of right buttock (disorder) | SNOMED#10878191000119102 |
| Gunshot wound of right buttock (disorder) | SNOMED#10878191000119102 |
| Gunshot wound of right foot (disorder) | SNOMED#10864011000119108 |
| Gunshot wound of right foot (disorder) | SNOMED#10864011000119108 |
| Gunshot wound of right foot (disorder) | SNOMED#10864011000119108 |
| Gunshot wound of right foot (disorder) | SNOMED#10864011000119108 |
| Gunshot wound of right foot (disorder) | SNOMED#10864011000119108 |
| Gunshot wound of right foot (disorder) | SNOMED#10864011000119108 |
| Gunshot wound of right foot (disorder) | SNOMED#10864011000119108 |
| Gunshot wound of right foot (disorder) | SNOMED#10864011000119108 |
| Gunshot wound of right foot (disorder) | SNOMED#10864011000119108 |
| Gunshot wound of right foot (disorder) | SNOMED#10864011000119108 |
| Gunshot wound of right foot (disorder) | SNOMED#10864011000119108 |
| Gunshot wound of right foot (disorder) | SNOMED#10864011000119108 |
| Gunshot wound of right foot (disorder) | SNOMED#10864011000119108 |
| Gunshot wound of right foot (disorder) | SNOMED#10864011000119108 |
| Gunshot wound of right foot (disorder) | SNOMED#10864011000119108 |
| Gunshot wound of right forearm (disorder) | SNOMED#10950071000119105 |
| Gunshot wound of right forearm (disorder) | SNOMED#10950071000119105 |
| Gunshot wound of right forearm (disorder) | SNOMED#10950071000119105 |
| Gunshot wound of right forearm (disorder) | SNOMED#10950071000119105 |
| Gunshot wound of right forearm (disorder) | SNOMED#10950071000119105 |
| Gunshot wound of right forearm (disorder) | SNOMED#10950071000119105 |
| Gunshot wound of right hand (disorder) | SNOMED#10950191000119107 |
| Gunshot wound of right hand (disorder) | SNOMED#10950191000119107 |
| Gunshot wound of right hand (disorder) | SNOMED#10950191000119107 |
| Gunshot wound of right hand (disorder) | SNOMED#10950191000119107 |
| Gunshot wound of right hand (disorder) | SNOMED#10950191000119107 |
| Gunshot wound of right hand (disorder) | SNOMED#10950191000119107 |
| Gunshot wound of right hand (disorder) | SNOMED#10950191000119107 |
| Gunshot wound of right hand (disorder) | SNOMED#10950191000119107 |
| Gunshot wound of right hand (disorder) | SNOMED#10950191000119107 |
| Gunshot wound of right hand (disorder) | SNOMED#10950191000119107 |
| Gunshot wound of right hand (disorder) | SNOMED#10950191000119107 |
| Gunshot wound of right hand (disorder) | SNOMED#10950191000119107 |
| Gunshot wound of right hand (disorder) | SNOMED#10950191000119107 |
| Gunshot wound of right hand (disorder) | SNOMED#10950191000119107 |
| Gunshot wound of right hand (disorder) | SNOMED#10950191000119107 |
| Gunshot wound of right hand (disorder) | SNOMED#10950191000119107 |
| Gunshot wound of right hand (disorder) | SNOMED#10950191000119107 |
| Gunshot wound of right hand (disorder) | SNOMED#10950191000119107 |
| Gunshot wound of right hand (disorder) | SNOMED#10950191000119107 |
| Gunshot wound of right hand (disorder) | SNOMED#10950191000119107 |
| Gunshot wound of right hand (disorder) | SNOMED#10950191000119107 |
| Gunshot wound of right hand (disorder) | SNOMED#10950191000119107 |
| Gunshot wound of right hand (disorder) | SNOMED#10950191000119107 |
| Gunshot wound of right hip region (disorder) | SNOMED#10861651000119101 |
| Gunshot wound of right hip region (disorder) | SNOMED#10861651000119101 |
| Gunshot wound of right hip region (disorder) | SNOMED#10861651000119101 |
| Gunshot wound of right hip region (disorder) | SNOMED#10861651000119101 |
| Gunshot wound of right hip region (disorder) | SNOMED#10861651000119101 |
| Gunshot wound of right hip region (disorder) | SNOMED#10861651000119101 |
| Gunshot wound of right knee region (disorder) | SNOMED#10862091000119107 |
| Gunshot wound of right knee region (disorder) | SNOMED#10862091000119107 |
| Gunshot wound of right knee region (disorder) | SNOMED#10862091000119107 |
| Gunshot wound of right knee region (disorder) | SNOMED#10862091000119107 |
| Gunshot wound of right knee region (disorder) | SNOMED#10862091000119107 |
| Gunshot wound of right knee region (disorder) | SNOMED#10862091000119107 |
| Gunshot wound of right lower leg (disorder) | SNOMED#10864081000119102 |
| Gunshot wound of right lower leg (disorder) | SNOMED#10864081000119102 |
| Gunshot wound of right lower leg (disorder) | SNOMED#10864081000119102 |
| Gunshot wound of right lower leg (disorder) | SNOMED#10864081000119102 |
| Gunshot wound of right lower leg (disorder) | SNOMED#10864081000119102 |
| Gunshot wound of right lower leg (disorder) | SNOMED#10864081000119102 |
| Gunshot wound of right shoulder region (disorder) | SNOMED#10939701000119109 |
| Gunshot wound of right shoulder region (disorder) | SNOMED#10939701000119109 |
| Gunshot wound of right shoulder region (disorder) | SNOMED#10939701000119109 |
| Gunshot wound of right shoulder region (disorder) | SNOMED#10939701000119109 |
| Gunshot wound of right shoulder region (disorder) | SNOMED#10939701000119109 |
| Gunshot wound of right shoulder region (disorder) | SNOMED#10939701000119109 |
| Gunshot wound of right thigh (disorder) | SNOMED#10861891000119105 |
| Gunshot wound of right thigh (disorder) | SNOMED#10861891000119105 |
| Gunshot wound of right thigh (disorder) | SNOMED#10861891000119105 |
| Gunshot wound of right thigh (disorder) | SNOMED#10861891000119105 |
| Gunshot wound of right thigh (disorder) | SNOMED#10861891000119105 |
| Gunshot wound of right thigh (disorder) | SNOMED#10861891000119105 |
| Gunshot wound of right upper arm (disorder) | SNOMED#10907441000119101 |
| Gunshot wound of right upper arm (disorder) | SNOMED#10907441000119101 |
| Gunshot wound of right upper arm (disorder) | SNOMED#10907441000119101 |
| Gunshot wound of right upper arm (disorder) | SNOMED#10907441000119101 |
| Gunshot wound of right upper arm (disorder) | SNOMED#10907441000119101 |
| Gunshot wound of right upper arm (disorder) | SNOMED#10907441000119101 |
| Gunshot wound of right upper arm (disorder) | SNOMED#10907441000119101 |
| Gunshot wound of upper limb (disorder) | SNOMED#23091000175103 |
| Gunshot wound of upper limb (disorder) | SNOMED#23091000175103 |
| Gunshot wound of upper limb (disorder) | SNOMED#23091000175103 |
| Injury due to airgun pellet (disorder) | SNOMED#243009001 |
| Injury due to airgun pellet (disorder) | SNOMED#243009001 |
| Injury due to airgun pellet (disorder) | SNOMED#243009001 |
| Injury due to airgun pellet (disorder) | SNOMED#243009001 |
| Injury due to airgun pellet (disorder) | SNOMED#243009001 |
| Injury due to airgun pellet (disorder) | SNOMED#243009001 |
| Injury due to airgun pellet (disorder) | SNOMED#243009001 |
| Injury due to airgun pellet (disorder) | SNOMED#243009001 |
| Injury due to airgun pellet (disorder) | SNOMED#243009001 |
| Injury due to airgun pellet (disorder) | SNOMED#243009001 |
| Injury due to airgun pellet (disorder) | SNOMED#243009001 |
| Injury due to airgun pellet (disorder) | SNOMED#243009001 |
| Injury due to airgun pellet (disorder) | SNOMED#243009001 |
| Injury due to airgun pellet (disorder) | SNOMED#243009001 |
| Injury due to airgun pellet (disorder) | SNOMED#243009001 |
| Injury due to airgun pellet (disorder) | SNOMED#243009001 |
| Injury due to airgun pellet (disorder) | SNOMED#243009001 |
| Injury due to airgun pellet (disorder) | SNOMED#243009001 |
| Injury due to airgun pellet (disorder) | SNOMED#243009001 |
| Injury due to airgun pellet (disorder) | SNOMED#243009001 |
| Injury due to bullet (disorder) | SNOMED#243000002 |
| Injury due to bullet (disorder) | SNOMED#243000002 |
| Injury due to bullet (disorder) | SNOMED#243000002 |
| Injury due to bullet (disorder) | SNOMED#243000002 |
| Injury due to bullet (disorder) | SNOMED#243000002 |
| Injury due to bullet (disorder) | SNOMED#243000002 |
| Injury due to bullet (disorder) | SNOMED#243000002 |
| Injury due to bullet (disorder) | SNOMED#243000002 |
| Injury due to bullet (disorder) | SNOMED#243000002 |
| Injury due to bullet (disorder) | SNOMED#243000002 |
| Injury due to legal intervention by firearm (disorder) | SNOMED#219257002 |
| Injury due to legal intervention by firearm (disorder) | SNOMED#219257002 |
| Injury due to legal intervention by firearm (disorder) | SNOMED#219257002 |
| Injury due to legal intervention by firearm (disorder) | SNOMED#219257002 |
| Injury due to legal intervention by firearm (disorder) | SNOMED#219257002 |
| Injury due to legal intervention by firearm (disorder) | SNOMED#219257002 |
| Injury due to legal intervention by firearm (disorder) | SNOMED#219257002 |
| Injury due to legal intervention by firearm (disorder) | SNOMED#219257002 |
| Injury due to legal intervention by firearm (disorder) | SNOMED#219257002 |
| Injury due to legal intervention by firearm (disorder) | SNOMED#219257002 |
| Injury due to legal intervention by firearm (disorder) | SNOMED#219257002 |
| Injury due to legal intervention by firearm (disorder) | SNOMED#219257002 |
| Injury due to legal intervention by firearm (disorder) | SNOMED#219257002 |
| Injury due to legal intervention by firearm (disorder) | SNOMED#219257002 |
| Injury due to legal intervention by firearm (disorder) | SNOMED#219257002 |
| Injury due to legal intervention by firearm (disorder) | SNOMED#219257002 |
| Injury due to legal intervention by firearm (disorder) | SNOMED#219257002 |
| Injury due to legal intervention by firearm (disorder) | SNOMED#219257002 |
| Injury due to legal intervention by firearm (disorder) | SNOMED#219257002 |
| Injury due to legal intervention by firearm (disorder) | SNOMED#219257002 |
| Injury due to legal intervention by firearm (disorder) | SNOMED#219257002 |
| Injury due to legal intervention by firearm (disorder) | SNOMED#219257002 |
| Injury due to legal intervention by firearm (disorder) | SNOMED#219257002 |
| Injury due to legal intervention by firearm (disorder) | SNOMED#219257002 |
| Injury due to legal intervention by firearm (disorder) | SNOMED#219257002 |
| Injury due to legal intervention by firearm (disorder) | SNOMED#219257002 |
| Injury due to legal intervention by firearm (disorder) | SNOMED#219257002 |
| Injury due to legal intervention by firearm (disorder) | SNOMED#219257002 |
| Injury due to legal intervention by firearm (disorder) | SNOMED#219257002 |
| Injury due to legal intervention by firearm (disorder) | SNOMED#219257002 |
| Injury due to legal intervention by firearm (disorder) | SNOMED#219257002 |
| Injury due to legal intervention by firearm (disorder) | SNOMED#219257002 |
| Injury due to legal intervention by firearm (disorder) | SNOMED#219257002 |
| Injury due to legal intervention by firearm (disorder) | SNOMED#219257002 |
| Injury due to legal intervention by firearm (disorder) | SNOMED#219257002 |
| Injury due to legal intervention by firearm (disorder) | SNOMED#219257002 |
| Injury due to legal intervention by machine gun (disorder) | SNOMED#219258007 |
| Injury due to legal intervention by machine gun (disorder) | SNOMED#219258007 |
| Injury due to legal intervention by machine gun (disorder) | SNOMED#219258007 |
| Injury due to legal intervention by machine gun (disorder) | SNOMED#219258007 |
| Injury due to legal intervention by machine gun (disorder) | SNOMED#219258007 |
| Injury due to legal intervention by machine gun (disorder) | SNOMED#219258007 |
| Injury due to legal intervention by machine gun (disorder) | SNOMED#219258007 |
| Injury due to legal intervention by machine gun (disorder) | SNOMED#219258007 |
| Injury due to legal intervention by machine gun (disorder) | SNOMED#219258007 |
| Injury due to legal intervention by machine gun (disorder) | SNOMED#219258007 |
| Injury due to legal intervention by machine gun (disorder) | SNOMED#219258007 |
| Injury due to legal intervention by machine gun (disorder) | SNOMED#219258007 |
| Injury due to legal intervention by revolver (disorder) | SNOMED#219259004 |
| Injury due to legal intervention by rifle bullet (disorder) | SNOMED#219260009 |
| Injury due to legal intervention by rifle bullet (disorder) | SNOMED#219260009 |
| Injury due to legal intervention by rifle bullet (disorder) | SNOMED#219260009 |
| Injury due to legal intervention by rifle bullet (disorder) | SNOMED#219260009 |
| Injury due to legal intervention by rifle bullet (disorder) | SNOMED#219260009 |
| Injury due to legal intervention by rifle bullet (disorder) | SNOMED#219260009 |
| Injury due to legal intervention by rifle bullet (disorder) | SNOMED#219260009 |
| Injury due to legal intervention by rifle bullet (disorder) | SNOMED#219260009 |
| Injury due to legal intervention by rifle bullet (disorder) | SNOMED#219260009 |
| Injury due to legal intervention by rifle bullet (disorder) | SNOMED#219260009 |
| Injury due to legal intervention by rifle bullet (disorder) | SNOMED#219260009 |
| Injury due to legal intervention by rifle bullet (disorder) | SNOMED#219260009 |
| Injury due to legal intervention by rubber bullet (disorder) | SNOMED#219261008 |
| Injury due to legal intervention by rubber bullet (disorder) | SNOMED#219261008 |
| Injury due to legal intervention by rubber bullet (disorder) | SNOMED#219261008 |
| Injury due to legal intervention by rubber bullet (disorder) | SNOMED#219261008 |
| Injury due to legal intervention by rubber bullet (disorder) | SNOMED#219261008 |
| Injury due to legal intervention by rubber bullet (disorder) | SNOMED#219261008 |
| Injury due to legal intervention by rubber bullet (disorder) | SNOMED#219261008 |
| Injury due to legal intervention by rubber bullet (disorder) | SNOMED#219261008 |
| Injury due to legal intervention by rubber bullet (disorder) | SNOMED#219261008 |
| Injury due to legal intervention by rubber bullet (disorder) | SNOMED#219261008 |
| Injury due to legal intervention by rubber bullet (disorder) | SNOMED#219261008 |
| Injury due to legal intervention by rubber bullet (disorder) | SNOMED#219261008 |
| Injury due to low velocity bullet (disorder) | SNOMED#243004006 |
| Injury due to low velocity bullet (disorder) | SNOMED#243004006 |
| Injury due to low velocity bullet (disorder) | SNOMED#243004006 |
| Injury due to low velocity bullet (disorder) | SNOMED#243004006 |
| Injury due to low velocity bullet (disorder) | SNOMED#243004006 |
| Injury due to low velocity bullet (disorder) | SNOMED#243004006 |
| Injury due to rubber bullet (disorder) | SNOMED#243010006 |
| Injury due to shotgun pellets (disorder) | SNOMED#243006008 |
| Injury due to shotgun pellets (disorder) | SNOMED#243006008 |
| Injury due to shotgun pellets (disorder) | SNOMED#243006008 |
| Injury due to shotgun pellets (disorder) | SNOMED#243006008 |
| Injury due to shotgun pellets (disorder) | SNOMED#243006008 |
| Injury due to shotgun pellets (disorder) | SNOMED#243006008 |
| Injury of unknown intent by handgun (disorder) | SNOMED#219335006 |
| Injury of unknown intent by handgun (disorder) | SNOMED#219335006 |
| Injury of unknown intent by handgun (disorder) | SNOMED#219335006 |
| Injury of unknown intent by handgun (disorder) | SNOMED#219335006 |
| Injury of unknown intent by handgun (disorder) | SNOMED#219335006 |
| Injury of unknown intent by handgun (disorder) | SNOMED#219335006 |
| Injury of unknown intent by handgun (disorder) | SNOMED#219335006 |
| Injury of unknown intent by handgun (disorder) | SNOMED#219335006 |
| Injury of unknown intent by handgun (disorder) | SNOMED#219335006 |
| Injury of unknown intent by handgun (disorder) | SNOMED#219335006 |
| Injury of unknown intent by handgun (disorder) | SNOMED#219335006 |
| Injury of unknown intent by handgun (disorder) | SNOMED#219335006 |
| Injury of unknown intent by handgun (disorder) | SNOMED#219335006 |
| Injury of unknown intent by handgun (disorder) | SNOMED#219335006 |
| Injury of unknown intent by handgun (disorder) | SNOMED#219335006 |
| Injury of unknown intent by hunting rifle (disorder) | SNOMED#219337003 |
| Injury of unknown intent by hunting rifle (disorder) | SNOMED#219337003 |
| Injury of unknown intent by hunting rifle (disorder) | SNOMED#219337003 |
| Injury of unknown intent by military firearm (disorder) | SNOMED#219338008 |
| Injury of unknown intent by military firearm (disorder) | SNOMED#219338008 |
| Injury of unknown intent by military firearm (disorder) | SNOMED#219338008 |
| Injury of unknown intent by shotgun (disorder) | SNOMED#219336007 |
| Injury of unknown intent by shotgun (disorder) | SNOMED#219336007 |
| Injury of unknown intent by shotgun (disorder) | SNOMED#219336007 |
| Suicide attempt by inducing lethal firearm response from law enforcement (event) | SNOMED#461261000124100 |
